# Parameterization of the Differences in Neural Oscillations Recorded by Wearable Magnetoencephalography for Chinese Semantic Cognition

**DOI:** 10.3390/biology14010091

**Published:** 2025-01-18

**Authors:** Xiaoyu Liang, Huanqi Wu, Yuyu Ma, Changzeng Liu, Xiaolin Ning

**Affiliations:** 1 School of Instrumentation Science and Optoelectronic Engineering, Beihang University, Beijing 100191, China; 2 Key Laboratory of Ultra-Weak Magnetic Field Measurement Technology, Ministry of Education, School of Instrumentation and Optoelectronic Engineering, Beihang University, Beijing 100191, China; 3 Institute of Large-Scale Scientific Facility and Centre for Zero Magnetic Field Science, Beihang University, Hangzhou 310051, China; 4 Hefei National Laboratory, Hefei 230088, China

**Keywords:** semantic processing, oscillations, OPM-MEG, cluster depth, parameterization

## Abstract

Neural oscillations observed during semantic processing embody the function of brain language processing. Precise parameterization of the differences in these oscillations across various semantics from a time–frequency perspective is pivotal for elucidating the mechanisms of brain language processing. The superlet transform and cluster depth test were used to compute the time–frequency representation of oscillatory difference (ODTFR) between neural activities recorded by optically pumped magnetometer-based magnetoencephalography (OPM-MEG) during processing congruent and incongruent Chinese semantics. Subsequently, ODTFR was parameterized based on the definition of local events. Finally, this study calculated the specific time–frequency values at which oscillation differences occurred in multiple auditory-language-processing regions. It was found that these oscillatory differences appeared in most regions and were mainly concentrated in the beta band. The average peak frequency of these oscillatory differences was 15.7 Hz, and the average peak time was 457 ms. These findings offer a fresh perspective on the neural mechanisms underlying the processing of distinct Chinese semantics and provide references and insights for analyzing language-related brain activities recorded by OPM-MEG.

## Simple Summary

This study used the superlet transform and the cluster depth test to compute the time–frequency representation (TFR) of the oscillatory differences between neural activities recorded by magnetoencephalography with optically pumped magnetometers while participants were listening to congruent and incongruent Chinese semantics. Then, the differences were parameterized based on the definition of local events. The results showed the TFRs of the differences in oscillatory activity occurring during various semantic processing tasks. The specific times, frequencies, and brain regions in which these differences occurred were demonstrated in detail. These results revealed the specific manifestations of the differences in neural oscillation activities during the cognition of semantically congruent and incongruent stimuli, which also revealed the potential causes of the differences in N400m neural activity and mismatch activities from the perspective of neural oscillations.

## 1. Introduction

Semantic cognition constitutes a crucial aspect of the language function of the human brain [1]. Semantic cognition refers to acquiring, storing, retrieving, and using knowledge about word and concept meanings and their relationships. The N400 component, which reflects semantic processing and is known as N400m in magnetoencephalography (MEG), peaks at approximately 400 ms after stimulus onset [2]. The ability to distinguish congruent and incongruent semantics is an important indicator of linguistic processing [3,4,5]. Congruent semantics refers to a situation in language where the meaning of words, phrases, or sentences aligns with preexisting knowledge, expectations, and semantic associations [2]. Incongruent semantics occurs when the meaning of words, phrases, or sentences deviates from our normal expectations and semantic associations. It creates a sense of conflict in the language [5]. Neural activity patterns exhibit differences in processing these two types of semantics, and this has been evidenced by non-invasive neuroscientific techniques such as electroencephalography (EEG) and magnetoencephalography (MEG) with superconducting quantum interference device (SQUID-MEG). In addition, some differences occur at N400m between congruent and incongruent semantics in the event-related field (ERF), namely, mismatch negativity (MMN) [6,7,8]. The amplitude of N400m observed in the left hemisphere significantly increases during processing incongruent semantics compared to congruent ones [9,10,11,12]. Optically pumped magnetometer (OPM)-based MEG (OPM-MEG) in the form of a wearable brain magnetic measurement technique [13] offers better source localization compared to EEG [14,15] and greater flexibility compared to SQUID-MEG [16,17]. In unrestricted conditions, it holds great potential for application [13,18] and provides a broader perspective for exploring the mechanisms of semantic processing in the brain. Wu et al. [2,19] demonstrated the effectiveness of OPM-MEG in recording neural activities and time-domain differences during the processing of congruent and incongruent semantics via a multivariate analysis from a temporal perspective.

Neural oscillation is an important aspect reflecting neural function [20]. The time–frequency features of oscillations are closely related to physiological activity states across different cognitive tasks [21,22]. Research has demonstrated alterations in the energy of beta- and alpha-band neural oscillations during semantic cognition activities, which is in temporal correlation with N400m [11,23]. Semantic processing typically occurs within a few hundred milliseconds [24]. Furthermore, oscillations across various frequency bands serve distinct roles in different brain regions within short durations [25,26]. It is essential to employ high-resolution time–frequency analysis methodologies to accurately capture the instantaneous characteristics of oscillatory activity during semantic processing, including specific frequencies and temporal occurrences on a single-trial basis. In the studies mentioned above, the time–frequency analysis (TFA) methods used for MEG mainly include short-time Fourier transform (STFT), wavelet transform (WT), and so on. The superlet transform (SLT) method proposed by Moca has very high time–frequency resolution for neuro-electrophysiological signals [27], and it exhibits superior performance in both the temporal and frequency resolution of neural activities compared to the most commonly used STFT, WT, and so on [27,28]. The SLT can effectively represent the instantaneous oscillatory activities during cognitive processes. Therefore, this paper employs the SLT to calculate the time–frequency representation (TFR) of neural activities.

Furthermore, the differences between neural activities associated with the processing of congruent versus incongruent semantic information are manifested through alterations in neural oscillations [29,30,31]. When listening to semantically congruent sentences, alpha-band neural oscillations within the temporoparietal cortex not only significantly intensify but also exhibit higher coherence between trials [32]. Moreover, compared with semantically incongruent stimuli, semantically congruent stimuli exhibit an increase in beta band power within 200–400 milliseconds [33,34]. These time–frequency differences in neural oscillatory activities underscore the sensitivity of the brain to congruent and incongruent semantics. Thus, precise calculation and analysis of the time–frequency differences in neural oscillations will facilitate a more reliable understanding of the neural mechanism of semantic processing.

It is essential to ensure that the identified differences are statistically significant, which can be solved by massively univariate tests, such as the cluster-based test [35], threshold-free cluster enhancement [36], and the cluster depth test [37]. Simultaneously, it is crucial to accurately compute the timing and frequency information of short-time changes in oscillations and ERF during semantic processing (such as the N400m [12] and mismatch activities [6]) from the perspective of oscillations. The cluster depth test proposed by Frossard aims to test the time-channel differences for EEG or MEG, allowing for a point-wise and channel-wise interpretation that can be used for determining the effects of timing [37]. Therefore, the cluster depth test method is applied to the TFR calculated by SLT in this paper, with ‘channel-wise’ replaced by ‘frequency-wise’. This approach will help to accurately represent the time–frequency differences in neural oscillatory activities.

Moreover, most studies have only focused on the time or frequency ranges of the neural oscillation differences associated with semantic processing [11,30,31] rather than on definitive numerical values. This may hinder an understanding of the precise characteristics of neural oscillations in semantic processing [38]. It is well established that external stimuli with fixed rhythms or durations can facilitate rehabilitation [39] and cognitive enhancement [40]. An approach to parameterization can achieve the calculation of definitive numerical values of those oscillation differences, which is used in this study.

In addition, Chinese is a language characterized by semantic features and that has the largest number of speakers globally [41]. It is of great significance to investigate the mechanism of semantic perception using Chinese semantic materials [41,42]. Consequently, the primary aims of this paper, in which neural activities are recorded by OPM-MEG during Chinese semantic processing, are as follows: (1) to illustrate how to use above-mentioned methods for the time–frequency representation and parameterization of oscillatory differences recorded by OPM-MEG for various semantic contents; and (2) to demonstrate the specific times, frequencies, and brain regions in which these differences in oscillatory activities occur during different semantic processing stages.

## 2. Materials and Methods

This section introduces the OPM-MEG data collection, data preprocessing, neural time series reconstruction, and the time–frequency representation and parameterization of the differences in neural activities in detail.

### 2.1. Subjects and Experiment

The data were obtained from a previously published Chinese auditory semantic OPM-MEG experiment [2]. The participants recruited for the experiment were required to be between 18 and 30 years old, have normal hearing, have no history of neurological or severe chronic diseases, have no cardiovascular or cerebrovascular diseases, have no metal materials implanted in their bodies, and have not participated in other similar studies. Furthermore, volunteers who failed to complete the research tasks as required or experienced serious adverse reactions were excluded from the study. Finally, the data of nine participants (mean age: 25.55 ± 2.58 years old; six males and three females) were analyzed in this study.

A traditional Chinese auditory final-verb N400m paradigm was used in this study. The semantics of the sentences are divided into two cases: (1) the final verb being congruous with semantic conditions; and (2) the last verb being incongruous with the semantic conditions [2]. Ethical approval for the experiment was issued by the Ethical Committee of Beihang University (No. BM20200175) on 20 July 2020.

### 2.2. Data Acquisition and Preprocessing

#### 2.2.1. Acquisition and Preprocessing of OPM-MEG Signals

OPM-MEG data were recorded by 36 second-generation magnetometers (Quspin Inc., Louisville, CO, USA), which covered the frontal, bilateral temporal, and parietal cortexes. The magnetic field perpendicular to the scalp was recorded. MEG signals and the triggers of semantic conditions were recorded with a PXI computer chassis (PXIC-7318C, ART Technology Inc., Beijing, China), and the positions and orientations of the OPM sensors in relation to the head of each subject were obtained with co-registration [2].

OPM-MEG data were preprocessed with 2–30 Hz bandpass filtering. A Picard independent component analysis [43] was used to remove artifacts arising from muscle activity, eye movement, and heartbeat. Multi-trial superposition and averaging of the preprocessed OPM-MEG signals were used to obtain the ERF and calculate the half-width of peaks, helping to determine the approximate time interval of the N400m component to be 200–400 ms.

#### 2.2.2. Source Reconstruction of ROI

The MRI data of the brains of all subjects were recorded by a Siemens MAGNETOM Prisma 3T scanner (Siemens AG, Munich, Germany) [2]. T1-weighted MRI scans were obtained using an MPRAGE sequence (TR, 2300 ms; TE, 3.03 ms; TI, 1100 ms; FA, 8; field of view, 256 × 256 × 192 mm; voxel size = 1.0 × 1.0 × 1.0 mm), which were pre-processed with Freesurfer [44] to extract the scalp surface and cortex. A forward model was constructed using MRI data and the positions and orientations of OPM sensors with MNE-Python [45]. Source spaces were established on the superior cortex in both hemispheres using recursively subdivided icosahedrons and downsampling [45]. A total of 5124 source spaces were set for each participant. This resulted in 5124 vertices in the white matter surfaces of the superior cortices in both hemispheres, whose neural time series could be reconstructed. A single-layer boundary element model (BEM) was constructed using the linear collocation method [45]. Subsequently, the source spatial information and the BEM model were utilized to construct the forward model.

**Figure 1:**
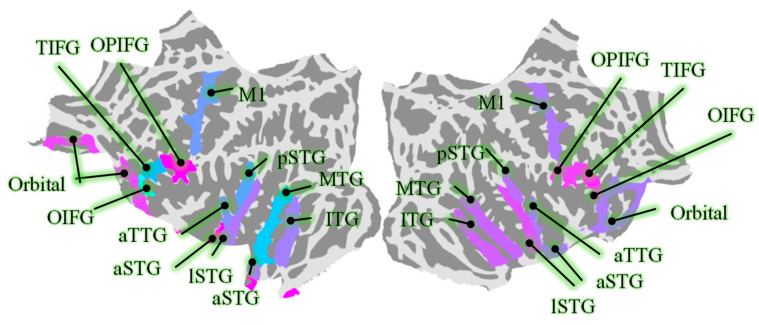
The selected ROIs, including middle temporal gyrus (MTG), precentral gyrus (M1), anterior transverse temporal gyrus (aSTG), orbital part of the inferior frontal gyrus (OIFG), opercular part of the inferior frontal gyrus (OPIFG), triangular part of the inferior frontal gyrus (TIFG), orbital gyri (Orbital), lateral aspect of the STG (lSTG), anterior of the STG (aSTG), posterior of the STG (pSTG), and inferior temporal gyrus (IFG).

According to previous studies [2,9], the time series of neural activity in regions of interest (ROIs) were obtained based on the aparc.a2009s atlas [46] and source reconstruction. The ROIs are shown in Figure 1. The time series of vertices in each ROI were constructed, and the numbers of vertices in the ROIs are shown in Table 1. The method of source reconstruction was dynamic statistical parametric mapping (dSPM), which integrates data from various imaging modalities along with prior anatomical and physiological knowledge to produce spatiotemporal estimations of brain activity [47]. These estimations are capable of achieving high levels of accuracy down to the millisecond scale. A focal source-level searchlight analysis was conducted on the ROIs to verify that the time-domain N400m spikes (400–800 ms) of the neural activity after source reconstruction still had excellent decoding performance for both congruent and incongruent semantic content. The specific methods and procedures are as described in Ref. [2].

### 2.3. The Time–Frequency Analysis of the Differences Between Neural Time Series Based on SLT and Cluster Depth Tests

Firstly, the time–frequency spectrum of the reconstructed neural signals for each ROI were calculated using SLT [27]. Subsequently, the cluster depth test [37] was employed to ascertain the *p* values for the differences in multi-trial TFRs of oscillatory activities at each vertex within the ROIs during the processing consistent and inconsistent semantics information. The initial number of cycles of SLT was set to 1. The minimum and maximum order of SLT were set to 1 and 30, respectively. A significance threshold of *p* < 0.05 was employed to obtain the TFR of oscillatory differences (ODTFR) for of all vertices in each ROI. Ultimately, the ODTFRs were averaged across all vertices in each ROI to provide a representation of the time–frequency differences in oscillatory activities between consistent and inconsistent semantic information processing within the ROIs. In the ODTFR, values greater than 0 indicate that the response under semantically inconsistent conditions is significantly stronger than that under semantically consistent conditions. Conversely, values less than 0 suggest that the response under semantically inconsistent conditions is significantly stronger. After obtaining the ODTFR for each ROI of each subject, the ODTFR features were parameterized.

### 2.4. Parameterization of Local Oscillatory Differences

**Figure 2:**
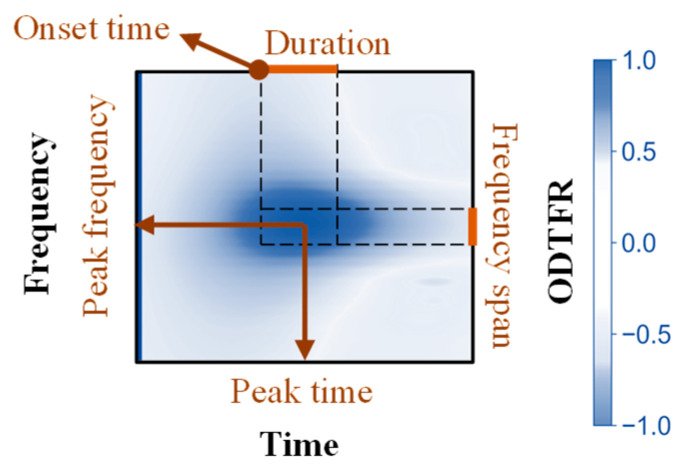
The definition of parameters in local peaks of ODTFR.

Time–frequency features for oscillatory differences in the ODTFRs were parameterized according to the definition of local events [48,49]. Firstly, multiple local peaks of ODTFRs were detected with the ‘scipy.ndimage’ library in Python. The frequencies and times corresponding to the extrema of these local peaks were designated as peak frequencies and peak times, respectively (see Figure 2).

For each local peak, time–frequency boundaries (coarse boundaries) were determined preliminarily. Additionally, the time (frequency) points at which the local peak power decreased to half of its maximum value (Half-Max) were computed. Subsequently, the boundaries of the local peaks were refined based on the Half-Max time (frequency) range. The refinement process involved the following steps: If the time (frequency) range of Half-Max fell within the coarse boundaries, the peak frequency was retained. If only one of the Half-Max boundaries exceeded the coarse boundaries, the within-bound boundary was retained, and the exceeding boundary was corrected to the Half-Max boundary. If both Half-Max boundaries exceeded the coarse boundaries, both boundaries were corrected to the Half-Max boundaries. Finally, for the different boundary correction scenarios, different methods were used to calculate the Full-Width at Half-Maximum (FWHM), which allowed for the calculation of duration and frequency span. Parameters and their definitions are shown in Table 2 and Figure 2.

## 3. Results

**Figure 3:**
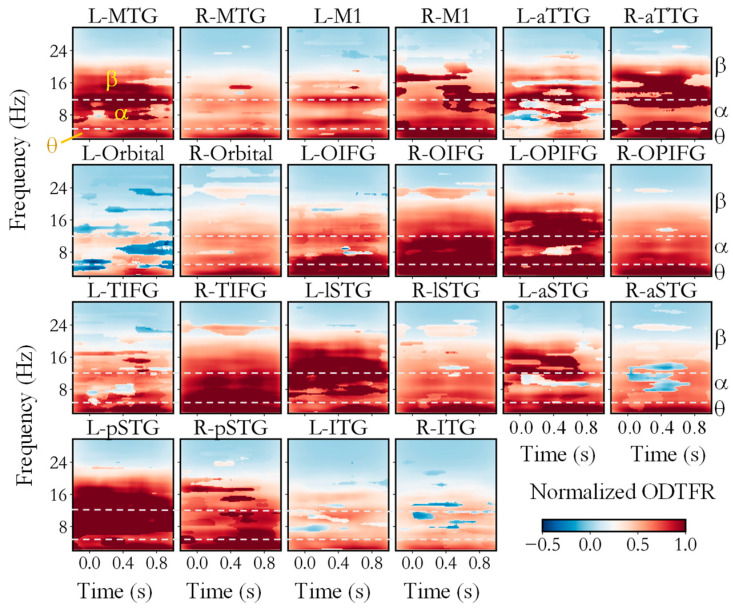
ODTFRs between congruent and incongruent semantics. Dark red and dark blue indicate significant time–frequency differences in neural activities under different semantic conditions.

Firstly, we utilized the SLT-CDT to calculate the time–frequency differences between neural activities during processing congruent and incongruent semantics within ROIs, as shown in Figure 3. Figure 3 shows the ODTFR of all ROIs, which were averaged across participants. Significant differences were apparent in the low-frequency, alpha, and low-beta bands (13–20 Hz) of the time–frequency spectrums across multiple cortical regions. Specifically, the low-frequency (<4 Hz) oscillations in L-MTG, R-MTG, L-M1, L-Orbital, and L-lSTG, predominantly occurred after 400 ms. The differences in low-frequency (<4 Hz) oscillations in the ITG were less pronounced compared to those in other ROIs. In other ROIs, distinct differences in neural oscillations occurred across the whole semantic-processing period. Significant differences in alpha-band oscillations were observed in L-MTG, L-M1, R-M1, aTTG, L-OIFG, R-OIFG, L-OPIFG, R-TIFG, L-lSTG, L-pSTG, and R-pSTG. Specifically, the oscillatory activities associated with semantic incongruence were notably stronger than those associated with semantic congruence. However, in L-Orbital, the oscillatory activities of semantically congruent situations were significantly stronger than those of semantically incongruent ones. As for the beta-band oscillations, strong differences were found in L-MTG, R-MTG (400–600 ms), R-M1, L-aTTG, R-aTTG, R-OIFG, L-OPIFG, L-lSTG, L-aSTG, L-pSTG, and R-pSTG. Slight differences in oscillatory activities also occurred in other brain regions. These results underscore the efficacy of OPM-MEG in capturing neural oscillations during the processing of Chinese semantics.

The ODTFRs roughly indicate the time–frequency range where differences occurred, and the parameters of the oscillatory differences from the ODTFRs are shown in Table 3, Table 4 and Table 5. It can be observed that differences in the beta band appeared in most regions.

Table 4 shows that differences in the alpha band only appeared in less than half of the regions. As shown in Table 3 and Table 4, both theta- and alpha-band oscillatory differences were observed in L-MTG, L-TIFG, L-aTTG, R-M1, R-TIFG, R-aTTG, and R-pSTG. As shown in Table 3 and Table 5, oscillatory differences occurred in both theta and beta bands in L-TIFG, L-aTTG, L-lSTG, L-pSTG, R-M1, R-TIFG, R-aTTG, and R-pSTG. As shown in Table 4 and Table 5, oscillatory differences in both alpha and theta bands occurred in L-TIFG, L-aTTG, L-lSTG, L-pSTG, R-M1, R-TIFG, R-aTTG, and R-pSTG. In addition, beta-band oscillatory differences also occurred in L-OPIFG, L-TIFG, L-aSTG, L-aTTG, R-M1, R-TIFG, R-aTTG, and R-pSTG, where alpha-band ones were not observed. In addition, oscillatory differences in L-MTG were only observed in the alpha band and theta band and not in beta band. The main difference between alpha- and beta-band oscillations occurred at the duration of L-OPIFG, L-TIFG, L-aTTG, R-M1, R-aTTG, and R-pSTG. The difference also occurred at the peak time and onset Time in L-OPIFG, L-TIFG, L-aSTG, R-TIFG, and R-pSTG. However, no significant oscillatory difference was identified in only R-ITG, according to Table 3, Table 4 and Table 5.

Furthermore, the peak times of these oscillatory activity differences mainly occurred between 200–600 ms, with an average peak time of 405 ms for theta-band oscillatory differences, 549 ms for alpha-band oscillatory differences, and 441 ms for beta-band oscillatory differences. The onset times of these oscillatory activity differences mainly occurred with an average peak time of 284 ms for theta-band oscillatory differences, 443 ms for alpha-band oscillatory differences, and 333 ms for beta-band oscillatory differences. The average peak frequency of all the differences was 15.7 Hz, and the average peak time was 457 ms. These results could be linked to the differences in the N400m component and MMN of the time-domain ERF during semantically consistent and inconsistent processing from a time–frequency domain perspective.

## 4. Discussion

### 4.1. Neural Activities During Semantic Processing

The neurophysiological activities in the brain when processing semantic information can reflect language perception mechanisms. The non-invasive OPM-MEG technique has opened new avenues for recording and analyzing neural activity during semantic processing [2,19]. This study introduced super-resolution SLT [27] combined with the cluster depth difference test [37] to explore the differences in short-time oscillatory activities when processing consistent and inconsistent Chinese semantic conditions. Significant differences were apparent in the low-frequency, alpha, and low-beta bands across multiple cortical regions (see Figure 3), similar to the findings of previous studies [11,31,32]. Furthermore, the analytical method effectively parameterized the differences in oscillatory activities across the different semantic processing tasks. By the time–frequency parameterization of the neural oscillatory activity differences, this study revealed the differences between the two types of semantic processing. For instance, differences in beta-band oscillations appeared in most ROIs (see Table 3), which is related to the crucial role of beta-band oscillations in cognition [11,50] and the peak time of the oscillatory differences mostly occurred at 200–600 ms. This phenomenon may also be the cause of difference in N400m and the MMN phenomenon, which has been proven to serve as a marker for human auditory semantic processing [6,8]. Previous studies have already demonstrated significant differences in neural activity (such as MMN) between healthy individuals and those with illnesses (e.g., schizophrenia) under different auditory trials [51,52]. Limited by practical conditions, the number of experimental samples in this study was restricted, and all samples were healthy adults. Therefore, the accumulation of more data from healthy individuals of different ages and patients in the future will help to further strengthen the conclusions of this study. Additionally, this research provides new references and insights for the application of the OPM-MEG in the recording and analysis of language-related brain activities.

Future research directions for advancing our understanding of neural activity during semantic processing and their contributions to the study of language-related brain functions are as follows. Firstly, employing MEG with other advanced neuroimaging techniques could precisely map the neural substrates involved in semantic processing in order to understand the exact neural mechanisms. Secondly, focusing on frequency-specific (such as beta-band) neural oscillations and conducting connectivity analysis could reveal how different brain regions communicate and coordinate during semantic processing, which could potentially allow for understanding the neural architecture supporting language functions and developing targeted therapeutic interventions for language disorders [2].

### 4.2. The Techniques for Analyzing Neural Activity During Semantic Processing

In this study, the statistical test method based on cluster depth contributed to the identification of short-duration oscillatory differences that consistently exist across multiple trials. However, it is important to note that burst oscillations occurring occasionally in individual trials may also carry physiological or neuroscientific significance related to language processing. Therefore, future research could explore additional methods such as PATPO [48] and BOSC [53] for parameterizing oscillatory activities in single trials, gaining a deeper understanding of the neural oscillatory mechanisms involved in semantic cognition.

Moreover, aperiodic activity, a neural activity that operates simultaneously with oscillatory activity, has shown potential correlations with cognitive functions [54,55]. The time–frequency characteristics of aperiodic components reflect the firing patterns of neuronal populations and the inhibition and enhancement of synaptic currents [56,57]. These activities can be parameterized using some techniques, such as FOOOF [56], ξ-π [56], and SPRiNT [57]. These advanced techniques will enable a better parameterization of aperiodic activities and transient oscillations, helping to reveal the mechanisms of neural activity during semantic processing from the perspectives of both aperiodic activities and oscillations.

Additionally, as a study driven by OPM-MEG data, the preprocessing method had an impact on the subsequent parameterization results. In particular, the filtering method employed in this paper is the widely used Infinite Impulse Response method [45]. Some newly developed methods, such as Complete Ensemble Empirical Mode Decomposition with Adaptive Noise [58], could contribute to improving the data denoising effect in the future, enabling a more accurate analysis of neural activities. For the time–frequency analysis, the SLT method used in this study has been proven to be more effective than most time–frequency analysis methods, such as the Choi–Williams and Wigner–Ville distributions and minimum mean cross-entropy [27]. With the advancement of technology and the expansion of application scenarios, this time–frequency analysis technique and the parameterization method used in this paper could be applied to a wider range of biological signal analysis scenarios, such as electromyogram signals [58].

In summary, four research directions could be expanded upon in the future: (1) more participants could be recruited, including healthy individuals and patients, to strengthen and expand the research presented in this paper; (2) by employing parameterizing methods, we could further explore the stable and short-duration neural activities during semantic processing; (3) the analysis of aperiodic activities could be introduced to gain a more comprehensive and in-depth understanding of the mechanisms of neural activity during semantic perception; and (4) more advanced processing methods could be employed to conduct more reliable analysis of more kinds of biosignals.

## 5. Conclusions

To analyze the differences in neural oscillations during Chinese semantic processing recorded by OPM-MEG, this paper introduced a super-resolution approach to the testing and parameterization of time–frequency differences. The time–frequency representations and parameters of the differences in oscillatory activity that occurred during various semantic processing tasks were calculated. The results elucidated the peak frequency, peak time, onset time, and duration of the differences in cortical oscillations when processing semantically consistent and inconsistent information. The results further revealed that the differences in neural oscillation activity were associated with the N400m and mismatch activities. This study can provide a reference for recording and analyzing language-related brain activity using OPM-MEG from the perspective of neural oscillations.

## Figures and Tables

**Table 1:** The numbers of vertices in ROIs (averaged across subjects).

ROI	Mean ± Std *	ROI	Mean ± Std *	ROI	Mean ± Std *	ROI	Mean ± Std *
L-MTG	42.7 ± 8.7	L-Orbital	36.6 ± 2.7	L-TIFG	18.6 ± 3.2	L-pSTG	24.6 ± 4.2
R-MTG	50.8 ± 9.6	R-Orbital	43.7 ± 3.4	R-TIFG	15.8 ± 2.9	R-pSTG	21.7 ± 4.2
L-M1	50.8 ± 7.1	L-OIFG	7.1 ± 2.4	L-lSTG	37.1 ± 5.3	L-ITG	40.6 ± 3.1
R-M1	54.6 ± 7.2	R-OIFG	5.3 ± 0.7	R-lSTG	34 ± 5.3	R-ITG	37.6 ± 4.1
L-aTTG	10.4 ± 1.2	L-OPIFG	25.3 ± 1.8	L-aSTG	15.3 ± 2.6		
R-aTTG	7.9 ± 0.6	R-OPIFG	26.1 ± 4.1	R-aSTG	20.6 ± 2.9		

* Std: Standard deviation.

**Table 2:** The definition of parameters of the time–frequency difference in oscillations.

Parameters	Definition
Peak Frequency	The frequency corresponding to local peaks in the ODTFR, where the difference in power is maximized.
Frequency span	The frequency span corresponding to the half-width of local peaks in the ODTFR and the frequency span over which the difference occurs.
Peak Time	The moment corresponding to local peaks in the ODTFR, where the difference in power is maximized.
Onset Time	The onset time corresponding to the half-width of local peaks in the ODTFR and the time at which the difference begins to appear.
Duration	The duration of time corresponding to the half-width of local peaks in the ODTFR and the duration over which the difference persists.

**Table 3:** Parameterized time–frequency differences in theta-band oscillations.

Band	ROI	Duration (ms)	Peak Frequency (Hz)	Peak Time (ms)	Onset Time (ms)
theta	L-MTG	400 ± 37	7.58 ± 0.18	499 ± 30	301 ± 34
	L-Orbital	300 ± 10	6 ± 0	371 ± 31	370 ± 31
	L-TIFG	116 ± 57	4.5 ± 1.13	234 ± 46	187 ± 23
	L-aTTG	566 ± 42	4.38 ± 0.57	536 ± 18	243 ± 21
	L-lSTG	146 ± 58	6.58 ± 0.08	502 ± 106	406 ± 103
	L-pSTG	230 ± 39	3.93 ± 0.44	363 ± 94	260 ± 111
	R-M1	156 ± 30	4 ± 0.09	276 ± 1	191 ± 17
	R-TIFG	228 ± 76	6.81 ± 0.36	444 ± 37	351 ± 62
	R-aTTG	312 ± 55	3.74 ± 0.21	497 ± 44	308 ± 51
	R-pSTG	135 ± 34	4.3 ± 0.18	324 ± 41	219 ± 9

**Table 4:** Parameterized time–frequency differences in alpha-band oscillations.

Band	ROI	Duration (ms)	Peak Frequency (Hz)	Peak Time (ms)	Onset Time (ms)
alpha	L-MTG	241 ± 67	8.56 ± 0.32	674 ± 84	627 ± 98
	L-OPIFG	351 ± 58	11.79 ± 0.44	520 ± 37	332 ± 47
	L-TIFG	92 ± 23	12.38 ± 0.07	626 ± 2	580 ± 7
	L-aSTG	413 ± 34	14.73 ± 0.28	444 ± 27	244 ± 13
	L-aTTG	19 ± 5	9.00 ± 1.15	926 ± 8	925 ± 8
	R-M1	262 ± 25	11.55 ± 0.3	536 ± 14	423 ± 15
	R-TIFG	328 ± 53	10.60 ± 0.49	318 ± 42	221 ± 30
	R-aTTG	463 ± 53	12.93 ± 0.71	363 ± 29	171 ± 29
	R-pSTG	154 ± 37	10.51 ± 0.15	535 ± 31	461 ± 42

**Table 5:** Parameterized time–frequency differences in beta-band oscillations.

Band	ROI	Duration (ms)	Peak Frequency (Hz)	Peak Time (ms)	Onset Time (ms)
beta	L-ITG	94 ± 52	25.94 ± 0.06	330 ± 44	293 ± 39
	L-M1	289 ± 45	24.10 ± 1.04	433 ± 39	289 ± 38
	L-OIFG	310 ± 11	26.75 ± 0.01	242 ± 3	241 ± 5
	L-OPIFG	84 ± 24	24.75 ± 1.46	707 ± 85	638 ± 91
	L-TIFG	249 ± 30	23.08 ± 0.93	436 ± 32	336 ± 33
	L-aSTG	371 ± 122	23.68 ± 2.21	499 ± 118	353 ± 144
	L-aTTG	128 ± 26	19.08 ± 1.17	563 ± 50	482 ± 51
	L-lSTG	57 ± 5	22.25 ± 0.01	620 ± 9	605 ± 10
	L-pSTG	241 ± 74	22.54 ± 0.08	275 ± 84	158 ± 96
	R-M1	154 ± 36	22.96 ± 0.68	472 ± 67	415 ± 68
	R-MTG	143 ± 28	21.23 ± 1.43	422 ± 27	372 ± 25
	R-OIFG	273 ± 61	23.53 ± 0.63	512 ± 65	406 ± 82
	R-OPIFG	378 ± 35	23.20 ± 0.09	352 ± 54	119 ± 22
	R-Orbital	314 ± 67	24.53 ± 0.87	422 ± 45	272 ± 49
	R-TIFG	314 ± 47	23.22 ± 0.41	489 ± 45	352 ± 57
	R-aSTG	374 ± 59	24.35 ± 0.52	478 ± 37	295 ± 55
	R-aTTG	257 ± 39	22.44 ± 0.98	283 ± 35	206 ± 32
	R-lSTG	445 ± 50	21.66 ± 0.42	474 ± 33	240 ± 35
	R-pSTG	254 ± 29	23.72 ± 0.70	374 ± 23	264 ± 22

## Data Availability

The raw data supporting the conclusions of this article will be made available by the authors on request.

## References

[1] Kutas M., Hillyard S.A. (1980). Reading Senseless Sentences: Brain Potentials Reflect Semantic Incongruity. Science.

[2] Wu H., Liang X., Wang R., Ma Y., Gao Y., Ning X. (2024). A Multivariate Analysis on Evoked Components of Chinese Semantic Congruity: An OP-MEG Study with EEG. Cereb. Cortex.

[3] Toro-Hernández F.D., Migeot J., Marchant N., Olivares D., Ferrante F., González-Gómez R., González Campo C., Fittipaldi S., Rojas-Costa G.M., Moguilner S. (2024). Neurocognitive Correlates of Semantic Memory Navigation in Parkinson’s Disease. npj Park. Dis..

[4] Irish M., Addis D.R., Hodges J.R., Piguet O. (2012). Considering the Role of Semantic Memory in Episodic Future Thinking: Evidence from Semantic Dementia. Brain.

[5] Broderick M.P., Anderson A.J., Lalor E.C. (2019). Semantic Context Enhances the Early Auditory Encoding of Natural Speech. J. Neurosci..

[6] Almeida V.N. (2021). Neurophysiological Basis of the N400 Deflection, from Mismatch Negativity to Semantic Prediction Potentials and Late Positive Components. Int. J. Psychophysiol..

[7] Heidlmayr K., Ferragne E., Isel F. (2021). Neuroplasticity in the Phonological System: The PMN and the N400 as Markers for the Perception of Non-Native Phonemic Contrasts by Late Second Language Learners. Neuropsychologia.

[8] Herman D., Baker S., Chow R., Cazes J., Alain C., Rosenbaum R.S. (2023). Mismatch Negativity as a Marker of Auditory Pattern Separation. Cereb. Cortex.

[9] Lau E.F., Phillips C., Poeppel D. (2008). A Cortical Network for Semantics: (De)Constructing the N400. Nat. Rev. Neurosci..

[10] Morett L.M., Landi N., Irwin J., McPartland J.C. (2020). N400 Amplitude, Latency, and Variability Reflect Temporal Integration of Beat Gesture and Pitch Accent during Language Processing. Brain Res..

[11] Wang L., Jensen O., van den Brink D., Weder N., Schoffelen J.-M., Magyari L., Hagoort P., Bastiaansen M. (2012). Beta Oscillations Relate to the N400m during Language Comprehension. Hum. Brain Mapp..

[12] Kutas M., Federmeier K.D. (2011). Thirty Years and Counting: Finding Meaning in the N400 Component of the Event Related Brain Potential (ERP). Annu. Rev. Psychol..

[13] Boto E., Holmes N., Leggett J., Roberts G., Shah V., Meyer S.S., Muñoz L.D., Mullinger K.J., Tierney T.M., Bestmann S. (2018). Moving Magnetoencephalography towards Real-World Applications with a Wearable System. Nature.

[14] Wang R., Liang X., Wu H., Yang Y., Zhao R., Gao Y., Ning X. (2024). Performance Evaluation of Interference Removal Methods Based on Subspace Projection with Wearable OPM-MEG. IEEE Trans. Instrum. Meas..

[15] An N., Cao F., Li W., Wang W., Xu W., Wang C., Gao Y., Xiang M., Ning X. (2022). Multiple Source Detection Based on Spatial Clustering and Its Applications on Wearable OPM-MEG. IEEE Trans. Bio. Med. Eng..

[16] Geller A.S., Teale P., Kronberg E., Ebersole J.S. (2023). Magnetoencephalography for Epilepsy Presurgical Evaluation. Curr. Neurol. Neurosci. Rep..

[17] Ma Y., Gao Y., Liang X., Wu H., Gao Z., Cao F., Li Y., Lu H., Liu C., Ning X. (2024). Evaluating the Performance of Optically Pumped Magnetometer Magnetoencephalography in Measuring Inter-Trial and Inter-Region Phase-Locking Value. Measurement.

[18] Brookes M.J., Leggett J., Rea M., Hill R.M., Holmes N., Boto E., Bowtell R. (2022). Magnetoencephalography with Optically Pumped Magnetometers (OPM-MEG): The next Generation of Functional Neuroimaging. Trends Neurosci..

[19] Wu H., Wang R., Ma Y., Liang X., Liu C., Yu D., An N., Ning X. (2024). Decoding N400m Evoked Component: A Tutorial on Multivariate Pattern Analysis for OP-MEG Data. Bioengineering.

[20] Doelling K.B., Assaneo M.F. (2021). Neural Oscillations Are a Start toward Understanding Brain Activity Rather than the End. PLoS Biol..

[21] Hincapié Casas A.S., Lajnef T., Pascarella A., Guiraud-Vinatea H., Laaksonen H., Bayle D., Jerbi K., Boulenger V. (2021). Neural Oscillations Track Natural but Not Artificial Fast Speech: Novel Insights from Speech-Brain Coupling Using MEG. NeuroImage.

[22] Düzel E., Penny W.D., Burgess N. (2010). Brain Oscillations and Memory. Curr. Opin. Neurobiol..

[23] Packard P.A., Steiger T.K., Fuentemilla L., Bunzeck N. (2020). Neural Oscillations and Event-Related Potentials Reveal How Semantic Congruence Drives Long-Term Memory in Both Young and Older Humans. Sci. Rep..

[24] Friederici A.D. (2002). Towards a Neural Basis of Auditory Sentence Processing. Trends Cogn. Sci..

[25] Mamashli F., Khan S., Obleser J., Friederici A.D., Maess B. (2019). Oscillatory Dynamics of Cortical Functional Connections in Semantic Prediction. Hum. Brain Mapp..

[26] Shahin A.J., Picton T.W., Miller L.M. (2009). Brain Oscillations during Semantic Evaluation of Speech. Brain Cogn..

[27] Moca V.V., Bârzan H., Nagy-Dăbâcan A., Mureșan R.C. (2021). Time-Frequency Super-Resolution with Superlets. Nat. Commun..

[28] Tripathi P.M., Kumar A., Kumar M., Komaragiri R.S. (2023). Automatic Seizure Detection and Classification Using Super-Resolution Superlet Transform and Deep Neural Network -A Preprocessing-Less Method. Comput. Methods Programs Biomed..

[29] Lewis A.G., Schoffelen J.-M., Hoffmann C., Bastiaansen M., Schriefers H. (2017). Discourse-Level Semantic Coherence Influences Beta Oscillatory Dynamics and the N400 during Sentence Comprehension. Lang. Cogn. Neurosci..

[30] Mai G., Minett J.W., Wang W.S.-Y. (2016). Delta, Theta, Beta, and Gamma Brain Oscillations Index Levels of Auditory Sentence Processing. NeuroImage.

[31] Strauß A., Kotz S.A., Scharinger M., Obleser J. (2014). Alpha and Theta Brain Oscillations Index Dissociable Processes in Spoken Word Recognition. NeuroImage.

[32] Noguchi Y. (2023). Combinatorial Binding of Semantic Information through the Sharing of Neural Oscillatory Signals. bioRxiv.

[33] Piai V., Roelofs A., van der Meij R. (2012). Event-Related Potentials and Oscillatory Brain Responses Associated with Semantic and Stroop-like Interference Effects in Overt Naming. Brain Res..

[34] Keitel A., Ince R.A.A., Gross J., Kayser C. (2017). Auditory Cortical Delta-Entrainment Interacts with Oscillatory Power in Multiple Fronto-Parietal Networks. NeuroImage.

[35] Maris E. (2012). Statistical Testing in Electrophysiological Studies: Statistical Testing in Electrophysiological Studies. Psychophysiology.

[36] Smith S.M., Nichols T.E. (2009). Threshold-Free Cluster Enhancement: Addressing Problems of Smoothing, Threshold Dependence and Localisation in Cluster Inference. NeuroImage.

[37] Frossard J., Renaud O. (2022). The Cluster Depth Tests: Toward Point-Wise Strong Control of the Family-Wise Error Rate in Massively Univariate Tests with Application to M/EEG. NeuroImage.

[38] Cross Z.R., Corcoran A.W., Schlesewsky M., Kohler M.J., Bornkessel-Schlesewsky I. (2022). Oscillatory and Aperiodic Neural Activity Jointly Predict Language Learning. J. Cogn. Neurosci..

[39] Jafari Z., Kolb B.E., Mohajerani M.H. (2020). Neural Oscillations and Brain Stimulation in Alzheimer’s Disease. Prog. Neurobiol..

[40] Calderone D.J., Lakatos P., Butler P.D., Castellanos F.X. (2014). Entrainment of Neural Oscillations as a Modifiable Substrate of Attention. Trends Cogn. Sci..

[41] Zhu Y., Xu M., Lu J., Hu J., Kwok V.P.Y., Zhou Y., Yuan D., Wu B., Zhang J., Wu J. (2022). Distinct Spatiotemporal Patterns of Syntactic and Semantic Processing in Human Inferior Frontal Gyrus. Nat. Hum. Behav..

[42] Zhang Q., Wang H., Luo C., Zhang J., Jin Z., Li L. (2019). The Neural Basis of Semantic Cognition in Mandarin Chinese: A Combined fMRI and TMS Study. Hum. Brain Mapp..

[43] Ablin P., Cardoso J.-F., Gramfort A. (2018). Faster Independent Component Analysis by Preconditioning With Hessian Approximations. IEEE Trans. Signal Process..

[44] Fischl B. (2012). FreeSurfer. NeuroImage.

[45] Gramfort A., Luessi M., Larson E., Engemann D.A., Strohmeier D., Brodbeck C., Parkkonen L., Hämäläinen M.S. (2014). MNE Software for Processing MEG and EEG Data. NeuroImage.

[46] Destrieux C., Fischl B., Dale A., Halgren E. (2010). Automatic Parcellation of Human Cortical Gyri and Sulci Using Standard Anatomical Nomenclature. NeuroImage.

[47] Dale A.M., Liu A.K., Fischl B.R., Buckner R.L., Belliveau J.W., Lewine J.D., Halgren E. (2000). Dynamic Statistical Parametric Mapping: Combining fMRI and MEG for High-Resolution Imaging of Cortical Activity. Neuron.

[48] Brady B., Bardouille T. (2022). Periodic/Aperiodic Parameterization of Transient Oscillations (PAPTO)–Implications for Healthy Ageing. NeuroImage.

[49] Shin H., Law R., Tsutsui S., Moore C.I., Jones S.R. (2017). The Rate of Transient Beta Frequency Events Predicts Behavior across Tasks and Species. eLife.

[50] Lundqvist M., Miller E.K., Nordmark J., Liljefors J., Herman P. (2024). Beta: Bursts of Cognition. Trends Cogn. Sci..

[51] Sauer A., Grent-’t-Jong T., Zeev-Wolf M., Singer W., Goldstein A., Uhlhaas P.J. (2023). Spectral and Phase-Coherence Correlates of Impaired Auditory Mismatch Negativity (MMN) in Schizophrenia: A MEG Study. Schizophr. Res..

[52] Hua J.P.Y., Roach B.J., Ford J.M., Mathalon D.H. (2023). Mismatch Negativity and Theta Oscillations Evoked by Auditory Deviance in Early Schizophrenia. Biol. Psychiatry Cogn. Neurosci. Neuroimaging.

[53] Hughes A.M., Whitten T.A., Caplan J.B., Dickson C.T. (2012). BOSC: A Better Oscillation Detection Method, Extracts Both Sustained and Transient Rhythms from Rat Hippocampal Recordings. Hippocampus.

[54] Hancock R., Pugh K.R., Hoeft F. (2017). Neural Noise Hypothesis of Developmental Dyslexia. Trends Cogn. Sci..

[55] Bertone A., Mottron L., Jelenic P., Faubert J. (2005). Enhanced and Diminished Visuo-Spatial Information Processing in Autism Depends on Stimulus Complexity. Brain.

[56] Donoghue T., Haller M., Peterson E.J., Varma P., Sebastian P., Gao R., Noto T., Lara A.H., Wallis J.D., Knight R.T. (2020). Parameterizing Neural Power Spectra into Periodic and Aperiodic Components. Nat. Neurosci..

[57] Wilson L.E., da Silva Castanheira J., Baillet S. (2022). Time-Resolved Parameterization of Aperiodic and Periodic Brain Activity. eLife.

[58] Elouaham S., Dliou A., Laaboubi M., Latif R., Elkamoun N., Zougagh H. (2020). Filtering and Analyzing Normal and Abnormal Electromyogram Signals. Indones. J. Electr. Eng. Comput. Sci..

